# Metformin Modulates T Cell Function and Alleviates Liver Injury Through Bioenergetic Regulation in Viral Hepatitis

**DOI:** 10.3389/fimmu.2021.638575

**Published:** 2021-04-21

**Authors:** Lanman Xu, Xiaofang Wang, Yan Chen, Lynn Soong, Yongping Chen, Jiyang Cai, Yuejin Liang, Jiaren Sun

**Affiliations:** ^1^Department of Infectious Diseases and Liver Diseases, Ningbo Medical Center Lihuili Hospital, Affiliated Lihuili Hospital of Ningbo University, Ningbo Institute of Innovation for Combined Medicine and Engineering, Ningbo, China; ^2^Department of Microbiology and Immunology, University of Texas Medical Branch, Galveston, TX, United States; ^3^Department of Infectious Diseases, Key Laboratory of Viral Hepatitis of Hunan, Xiangya Hospital, Central South University, Changsha, China; ^4^Department of Ophthalmology, University of Texas Medical Branch, Galveston, TX, United States; ^5^Department of Pathology, University of Texas Medical Branch, Galveston, TX, United States; ^6^Institute for Human Infections and Immunity, University of Texas Medical Branch, Galveston, TX, United States; ^7^Department of Infectious Diseases, The First Affiliated Hospital of Wenzhou Medical University, Zhejiang Provincial Key Laboratory for Accurate Diagnosis and Treatment of Chronic Liver Diseases, Wenzhou, China

**Keywords:** mTOR, T cell, mitochondria, metformin, viral hepatitis

## Abstract

Metformin is not only the first-line medication for the treatment of type 2 diabetes, but it is also effective as an anti-inflammatory, anti-oxidative and anti-tumor agent. However, the effect of metformin during viral hepatitis remains elusive. Using an adenovirus (Ad)-induced viral hepatitis mouse model, we found that metformin treatment significantly attenuated liver injury, with reduced serum aspartate transaminase (AST) and alanine transaminase (ALT) levels and liver histological changes, presumably *via* decreased effector T cell responses. We then demonstrated that metformin reduced mTORC1 activity in T cells from infected mice, as evidenced by decreased phosphorylation of ribosome protein S6 (p-S6). The inhibitory effects on the mTORC1 signaling by metformin was dependent on the tuberous sclerosis complex 1 (TSC1). Mechanistically, metformin treatment modulated the phosphorylation of dynamin-related protein 1 (Drp-1) and mitochondrial fission 1 protein (FIS1), resulting in increased mass in effector T cells. Moreover, metformin treatment promoted mitochondrial superoxide production, which can inhibit excessive T cell activation in viral hepatitis. Together, our results revealed a protective role and therapeutic potential of metformin against liver injury in acute viral hepatitis *via* modulating effector T cell activation *via* regulating the mTORC1 pathway and mitochondrial functions.

## Introduction

Metformin is a biguanide drug that has been used to treat type 2 diabetes mellitus and to help control blood glucose levels for more than 60 years (1, 2). No significant safety issues from long-term use of metformin have been identified for diabetes prevention (3), indicating that, as an affordable medicine, metformin is safe and effective. Recent studies indicate that metformin may have additional benefits in several other diseases, such as cancer, stroke, neurodegenerative diseases, obesity, metabolic syndrome, and cardiovascular diseases (1, 4–7). The function of metformin is closely linked to the mitogen-activated protein kinase and mechanistic target of rapamycin (mTOR) pathway (8–10). mTOR regulates cell growth, cell proliferation, cell motility, cell survival, protein synthesis, autophagy and transcription (11, 12). Signaling through mTOR regulates metabolism and is an important molecular connection between nutrient signals and the metabolic processes that are indispensable for cell growth and function (13).

Viral hepatitis is a major health concern globally and the main cause of hepatocellular carcinoma (14). Immune responses play a critical role in fulminant viral hepatitis (15), which can be observed after acute viral infection accompanied by strong (re)-activation of the immune response in some patients (16). Accumulating evidence has showed that metformin may have therapeutic potential in liver diseases (1), as metformin can improve the survival of diabetic liver cancer patients (17). In mice, metformin treatment showed preventative effects in liver carcinogenesis by downregulating the expression of lipogenic enzymes and lipogenesis (18). Results from human and animal studies also showed that the administration of metformin improves liver function in non-alcoholic fatty liver disease (NAFLD) subjects (19, 20). Metformin may also suppress hepatitis B virus and hepatitis C virus (HCV) replication *in vitro* (21, 22), potentially through a type I IFN-dependent mechanism (23). However, it remains unclear as to whether and how metformin directly modulates T cell responses in viral hepatitis.

In this study, we challenged mice with a hepatotropic adenovirus (Ad) type 5 (24), which can cause strong virus-specific T cell responses and subsequent liver damage. We found that metformin treatment significantly attenuated liver damage as judged by serum aspartate transaminase (AST) and alanine transaminase (ALT) levels, hepatic histological scores, and proinflammatory cytokine (IFN-γ, TNF-α and IL-2) production. Our *in vivo* and *in vitro* data further demonstrated that metformin restrained T cell activation, inhibited phosphorylation of S6 (p-S6) in the mTOR signaling pathway, reduced mitochondrial fission, but increased the production of superoxide in T cells. These results suggest that metformin can directly modulate T cell responses by remodeling mitochondrial dynamics.

## Materials and Methods

### Animals and Treatment

Female C57BL/6 (B6) and tuberous sclerosis 1 (TSC1) ^flox^ (#005680) mice were purchased from the Jackson Laboratory (Bar Harbor, ME). Metformin was purchased from Sigma-Aldrich (St. Louis, MO). Mice were orally pretreated with metformin (Sigma-Aldrich, St. Louis, MO, 250 mg/kg/day) for 1 week by gavage, followed by intravenous (i.v.) infection of adenovirus as described previously (25). Administration of metformin was continued for another 7 days. Normal saline was used as a control. All mice were euthanized at 7 days post-infection (dpi). Adenovirus carrying LacZ (AdLacZ) and Cre (AdCre), purchased from Vector Development Laboratory, Baylor College of Medicine, were used to infect B6 and TSCl ^flox^ mice in indicated experiments. The dose of 1.8 × 10^9^ pfu/mouse *via* i.v. injection route. All mice were maintained and bred under specific pathogen-free conditions in the animal facility at the University of Texas Medical Branch (UTMB). Seven- to twelve-week-old mice were used for all the experiments. All experiments were reviewed and approved by the Institutional Animal Care and Use Committees of the University of Texas Medical Branch.

### Detection of AdLacZ in Liver Sections

The detection of AdLacZ was performed as previously described (24). Briefly, following fixation with 0.5% glutaraldehyde for 30 min, frozen liver sections were incubated with 0.2 mg of X-gal (5-bromo-4-chloro-3-indolyl-β-d-galactopyranoside)/ml at 37°C for 120 min. Infected cells expressing β-gal activity were stained blue. Eight images of each liver section were randomly selected and captured with the Olympus BX51 microscope equipped with the Olympus DP70 video camera (Olympus Optical, Tokyo, Japan). The images were analyzed by Image-Pro Plus 6.0 software (Media Cybernetics, MD, USA). Infectivity of the hepatocytes was expressed as the percentage of positive staining areas.

### Antibodies (Abs) and Reagents

All fluorochrome-labeled mAbs and their corresponding isotype controls were purchased from Thermo Fisher Scientific (San Diego, CA), including PE-Cy7-conjugated anti-CD3 (17A2), Pacific Blue-conjugated anti-CD4 (RM4-5), APC-eFlour780-conjugated anti-CD4 (RM4-5), PerCp-Cy5.5-conjugated anti-CD8 (53-6.7), APC-eFlour780-conjugated anti-CD8 (53-6.7), PerCp-eFlour710-conjugated anti-TNF-α (MP6-XT22), APC-conjugated anti-IFN-*γ* (XMG1.2), PE- conjugated anti-CD44 (IM7), APC-conjugated anti-CD62L (MEL-14) and FITC-conjugated anti-IL-2 (JES6-5H4). Purified anti-CD16/32 (2.4G2), anti-CD3 (2C11) and anti-CD28 (37.51) were purchased from Biolegend (San Diego, CA). Mito Tracker Green and p-S6 Ribosomal Protein (Ser235/236) (D57.2.2E) Rabbit mAb (PE Conjugate) were purchased from Cell Signaling Technology (Beverly, MA). Mitochondrial superoxide indicator and mouse p-Drp-1 (Ser637) were purchased from Thermo Fisher Scientific (San Diego, CA). Mouse p-Drp-1 (Ser616) pAb was purchased from Abclonal Technology (Woburn, MA). Mouse FIS1 Ab was purchased from Proteintech (Rosemont, IL).

### Histological and Serum Biochemical Analysis

Liver specimens were fixed in 10% buffered formalin. Paraffin-embedded sections were stained with H&E for histological evaluation using a Histology Activity Index Score (HAI-Knodell score) (26). Serum was collected for AST and ALT measurement by an automatic biochemistry analyzer (Beckman Coulter) in the Department of Clinical Chemistry, UTMB.

### Isolation of Intrahepatic Lymphocytes (IHL)

IHL were isolated according to our previous method (24, 25). In brief, mouse liver was first perfused with PBS without calcium and magnesium. Liver tissue was collected and digested in RPMI 1640 containing collagenase IV (0.05%; Roche Applied Science, Indianapolis, IN) at 37˚C for 30 min. After digestion, cell suspension was passed through 70-mm nylon cell strainers to remove aggregates and yield single-cell suspensions. IHL were purified by centrifugation (400 g) at room temperature for 30 min over a 30/70% discontinuous Percoll gradient (Sigma-Aldrich St. Louis, MO). The IHL were collected from the interphase, thoroughly washed, and resuspended in complete RPMI 1640 containing 10% FBS (Hyclone, Logan, UT). The total numbers of IHL per liver were counted.

### Flow Cytometry Analysis

For surface staining, cells were first incubated with FcγR blocker (CD16/32) at room temperature for 5 min, followed by staining with fluorochrome-labeled Abs at 4°C for 30 min in dark. For intracellular staining, cells were incubated with PMA (50 ng/mL), ionomycin (750 ng/mL) and Brefeldin A (BD Biosciences) for 5 h. After incubation, cells were collected for surface staining, followed by fixation and permeabilization using a fixation/permeabilization kit (Thermo Fisher Scientific). Incubation of intracellular Abs was performed at 4°C for 45 min in dark. The phosflow experiments were performed according to the protocol from BD Biosciences. Briefly, the cells were stimulated with cytokines at the indicated times, followed by immediate fixation using a prewarmed Cytofix Fixation Buffer at 37°C for 12 min. The cells were permeabilized using chilled Perm Buffer III for 1 h on ice and were then washed and stained with Cell Signaling Technology phosflow Abs. Samples were processed on an LSRII FACS Fortessa and analyzed using FlowJo X software (Tree Star, Ashland, OR).

### Quantitative Reverse Transcriptase-PCR (qRT-PCR)

RNA was extracted using an RNeasy Mini Kit according to the instructions (Qiagen, Valencia, CA). The synthesis of cDNA was proceeded using an iScript Reverse Transcription Kit (Bio-Rad, Hercules, CA). cDNA was amplified in a 10-μl reaction mixture containing 5 μl of iTaq SYBR Green Supermix (Bio-Rad) and 5 μM each of gene-specific forward and reverse primers. The PCR assays were denatured for 30 s at 95°C, followed by 40 cycles of 15 s at 95°C, and 60 s at 60°C, utilizing the CFX96 Touch real-time PCR detection system (Bio-Rad). Relative quantitation of mRNA expression was calculated using the 2^-ΔΔCt^ method. The primers are as below. *GAPDH* Forward 5’ -TGGAAAGCTGTGGCGTGAT-3’, Reverse 5’ -TGCTTCACCACCTTCTTGAT-3’; *TSC1* Forward 5’ –ATGGCCCAGTTAGCCAACATT, Reverse 5’ – CAGAATTGAGGGACTCCTTGAAG.

### Measurement of Mitochondrial Mass and Superoxide

IHL and splenocytes were cultured in 48-well plates with metformin at different concentrations (0, 1, 5, 10 nM) for the indicated times (6 and 12 h). Cells were collected for surface staining, followed by incubation (37°C, 20 min) with 200 nM Mito Tracker Green and 5 μM MitoSOX for mitochondrial mass and superoxide, respectively. After incubation, cells were washed and resuspended in FACS buffer for analysis.

### CD8^+^ T Cell Purification

The spleen was mechanically dissociated and incubated with red blood cell lysis buffer (Sigma-Aldrich St. Louis, MO) to remove red blood cells. CD8^+^ T cells were purified using CD8a^+^ T Cell Isolation Kit (Miltenyi Biotech). The purity of CD8^+^ T cells was determined by flow cytometry and was higher than 95%.

### Western Blot Analysis and ELISA Assay

Cell protein was extracted using a RIPA lysis buffer (Cell Signaling Technology) in the presence of the phosphatase inhibitor cocktail (Thermo Fisher Scientific). Protein concentrations were measured using a BCA Protein Assay Kit (Pierce). Protein samples were separated by SDS-PAGE (4–15%) and electro-transferred onto a PVDF membrane, which was then blocked with 5% BSA for 60 min. The membrane was then incubated with primary Abs overnight at 4°C. After incubation, the membrane was washed 3 times with TBST, incubated with secondary Abs for 60 min at room temperature and developed using the ECL western blotting substrate reagent (Thermo Fisher Scientific). The signal intensity was analyzed by Image J and normalized to β-actin. Hepatic IL-2, TNF-α, and IFN-γ protein levels were assayed using specific ELISA kits (Biolegend) according to the manufacturer’s instructions.

### Statistical Analysis

The difference between two groups was determined using a two-tailed Student t test. One-way ANOVA was used for multiple-group comparisons, followed by Tukey’s multiple comparisons (GraphPad Software v7.0, San Diego, CA). *, **, *** or **** represent *p*-values<0.05, <0.01, <0.001 or <0.0001, respectively.

## Results

### Metformin Treatment Alleviates Liver Injury in Ad-Induced Viral Hepatitis

**Figure 1:**
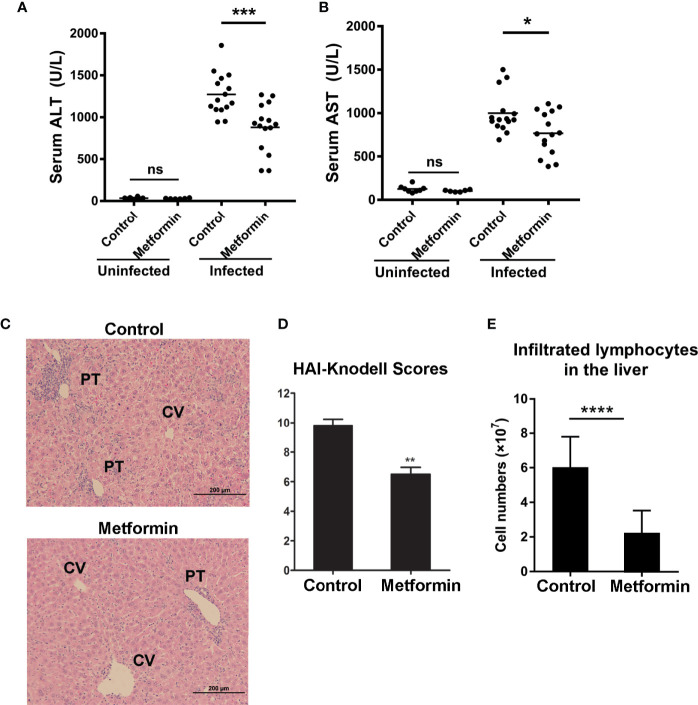
Metformin treatment alleviates liver injury in Ad-induced viral hepatitis. B6 mice were orally pretreated with metformin (250 mg/kg/day) for 1 week, followed by infection of adenovirus carrying LacZ (AdLacZ, 1.8 × 10^9^ pfu/mouse). Administration of metformin was continued for another 7 days. Normal Saline was used as a control. All mice were euthanized at 7 days post injection (7dpi). Uninfected mice were used as controls. **(A)** Serum ALT. **(B)** Serum AST. **(C)** Representative images of H&E staining for livers. PT, portal tracts; CV, central vein. **(D)** Liver pathological HAI-Knodell scores. **(E)** Numbers of intrahepatic lymphocytes. Two-tailed unpaired T test is used for ALT and AST statistical analysis. Mann-Whitney U test is used for liver pathological statistical analysis. *p < 0.05; **p < 0.01; ***p < 0.001; ****p < 0.0001.

To determine whether metformin protects mice from liver injury in viral hepatitis, we pretreated B6 mice with metformin (250 mg/kg/day) daily for 1 week before i.v. infection of AdLacZ (1.8 × 10^9^ pfu/mouse). Administration of metformin was continued for another 7 days. Serum ALT, AST and liver pathological changes at 7 dpi were used as readouts. Control animals developed prominent liver inflammation as measured by elevation of ALT and AST levels and considerable lymphocyte infiltration. Metformin pre-treatment significantly decreased ALT and AST levels, together with reduction of lymphocyte infiltration (**Figures 1A–C**). No difference of ALT and AST was observed in naïve mice with or without metformin treatment (**Figures 1A, B**). Histological evaluation confirmed lower liver pathological scores in the metformin-pretreated group compared with those in the control group (**Figure 1D**). The numbers of intrahepatic lymphocytes were also lower in metformin-treated mice compared with that of control mice (**Figure 1E**). When analyzed viral load by measuring the area with positive X-gal-staining, we found that metformin-pretreated mice had much less hepatic viral load than that of the control group (**Figure S1**). These results demonstrate that metformin protected mice from liver damage in Ad-induced viral hepatitis.

### Metformin Limits Pro-Inflammatory Cytokine Production From T Cells

**Figure 2:**
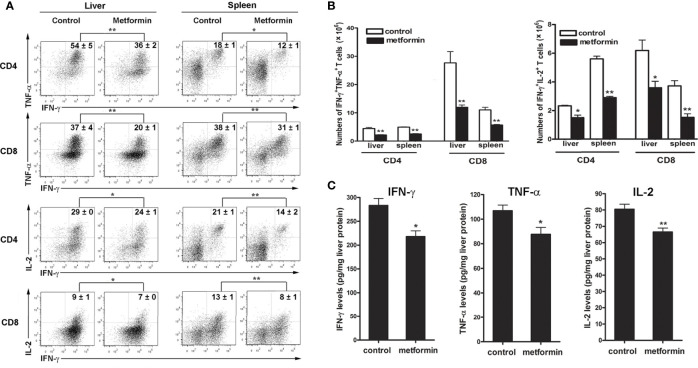
Metformin limits pro-inflammatory cytokine production from T cells. Mice were infected and treated with metformin as in **Figure 1**. **(A)** Lymphocytes from liver and spleen were stimulated with PMA and ionomycin in the presence of Golgi Stop for 5 h, followed by intracellular staining of IFN-γ, TNF-α and IL-2. **(B)** Numbers of IFN-γ^+^ TNF-α^+^ T cells and IFN-γ^+^ IL-2^+^ T cells in liver and spleen. **(C)** The protein levels of liver TNF-α, IFN-γ and IL-2 were detected by ELISA. The data are shown as mean ± SEM of n = 3-5 mice/group from single experiments representative of at least three experiments performed. Two-tailed unpaired T test was used for statistical analysis. *p < 0.05; **p < 0.01.

Hepatic infiltration and activation of T effector cells result in liver injury in a dose-dependent fashion (27). To determine whether metformin can regulate T cell functions, we evaluated splenic and intrahepatic T cell activation at 7 dpi and found fewer numbers of activated T cells (CD44^+^CD62L^-^) in the spleen of metformin-treated mice, accompanied with a decreased trend of activated T cells in the liver (**Figure S2**). More importantly, metformin treatment significantly decreased percentages of IFN-γ^+^ TNF-α^+^ and IFN-γ^+^ IL-2^+^ cells among CD4^+^ and CD8^+^ T cells in the liver and spleen (**Figure 2A**). As expected, the numbers of IFN-γ^+^ TNF-α^+^ and IFN-γ^+^IL-2^+^ T cells were also lower in the metformin group than those in the control group (**Figure 2B**). These results were further supported by decreased IFN-γ, TNF-α and IL-2 production in the liver of Ad-infected mice (**Figure 2C**).

**Figure 3:**
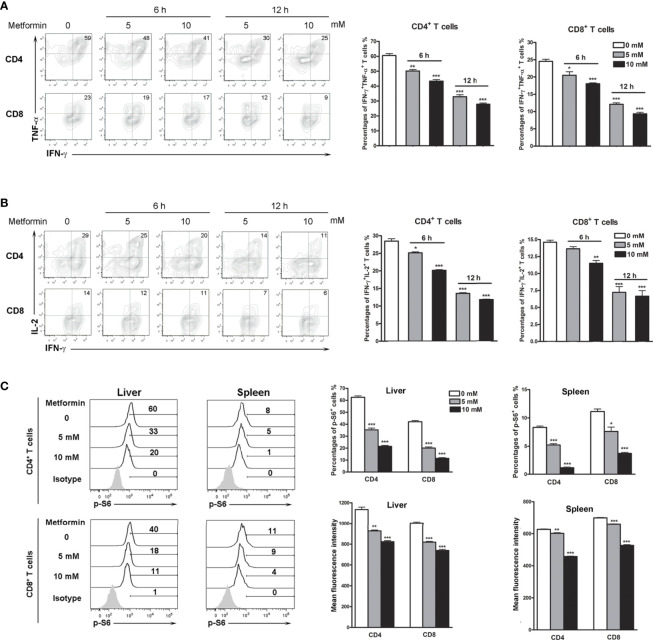
Metformin inhibits T cell activation and p-S6 *in vitro*. Mice were injected i.v. with 1.8 × 10^9^ pfu of AdLacZ and sacrificed at 7 dpi. Lymphocytes from liver and spleen were isolated and cultured with the indicated concentrations of metformin (0, 5 and 10 mM) for 6 and 12 h. Cells were stimulated with PMA and ionomycin in the presence of GolgiStop during the last 5 h, followed by intracellular staining of IFN-γ, TNF-α, IL-2 and p-S6. **(A)** Percentages of IFN-γ^+^ TNF-α^+^ T cells from livers. **(B)** Percentages of IFN-γ^+^ IL-2^+^ T cells from livers. **(C)** Percentages and mean fluorescence intensity (MFI) of p-S6^+^ in liver and spleen T cells. Data are representative of at least three independent experiments. Values are shown as mean ± SEM of n = 3-4 samples/group from single experiments representative of at least three experiments performed. Two-tailed unpaired T test was used for statistical analysis. *p < 0.05; **p < 0.01; ***p < 0.001.

To investigate if metformin can directly regulate T cell activities in Ad-infected mice, we isolated IHL from infected mice, followed by metformin treatment *in vitro*. We first performed a cytotoxic experiment to determine the dose of metformin that we will use in the following experiments. Our result showed that low doses of metformin (less than 10 mM) had no toxicity within 12 h *in vitro* (**Figure S3**). We further found that metformin inhibited T cell activation in dose- and time-dependent manners as evidenced by decreased percentages of IFN-γ^+^ TNF-α^+^ (**Figure 3A**) and IFN-γ^+^IL-2^+^ T cells (**Figure 3B**). The splenocytes from infected mice showed similar trends (**Figure S4**). Together, our results demonstrate that metformin down-regulates T cell activation *in vivo* and *in vitro*, limits intrahepatic infiltration, and inhibits inflammatory cytokine production in Ad-induced hepatitis.

### The TSC1-Dependent mTOR Signaling Pathways Regulated by Metformin

It is known that while the mTOR signaling pathway is critical for T cell activation and differentiation (28), mTOR is also a potential target of metformin (29, 30). We therefore asked whether metformin can regulate mTOR signaling in T cells of virus-infected mice. We then treated isolated lymphocytes with metformin *in vitro* and analyzed p-S6 levels. We found that metformin treatment down-regulated p-S6 in both splenocytes and IHL in a dose-dependent manner (**Figure 3C**).

**Figure 4:**
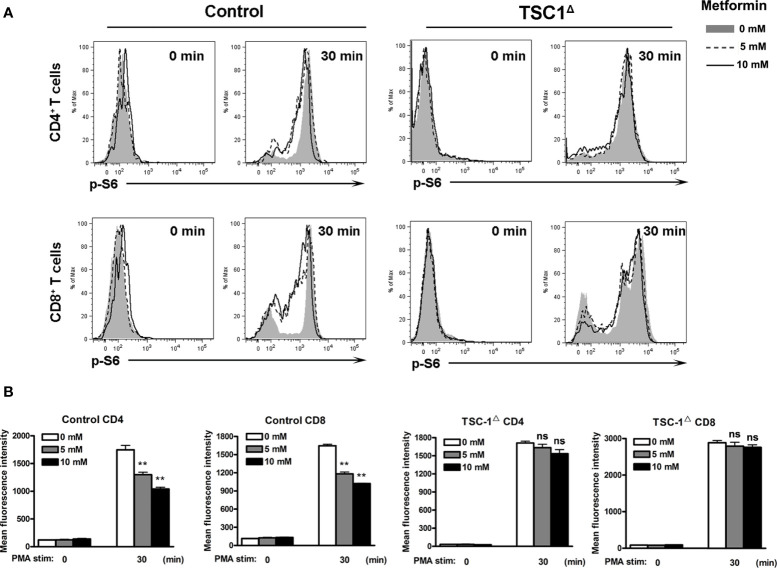
Metformin inhibits mTOR activity in T cells by a TSC1-dependent manner. TSC1^flox^ and control mice were injected i.v. with 1.8 × 10^9^ pfu of AdCre. Intrahepatic lymphocytes (IHL) were isolated at 7 dpi and cultured with the indicated concentrations of metformin (0, 5 and 10 mM) for 6 h. Cells were stimulated with PMA for 30 min, then fixed immediately and analyzed for p-S6 by flow cytometry. Unstimulated cells (0 min) were used as controls. **(A)** Representative images of p-S6^+^ T cells. **(B)** MFI of p-S6. Data are representative of at least three independent experiments. Values are shown as mean ± SEM of n = 3-4 samples/group from single experiments representative of at least three experiments performed. One-way ANOVA with Tukey’s multiple comparisons test was used. Metformin treatment groups (5 and 10 mM) were compared to the control group (0 mM). **p < 0.01. ns, no significant difference.

The TSC complex is known to be the upstream suppressor of mTOR complex 1 (mTORC1)-mediated pathway, playing regulatory roles in naive T cell quiescence (31). To investigate the mechanism of metformin in regulating the mTOR signaling pathway, we depleted liver TSC1 by infecting the TSC1^flox^ mice with AdCre, as we reported previously (32). The qPCR result of liver tissues showed the significant decreased expression of TSC1 in AdCre-infected TSC1^flox^ mice at 7 dpi (**Figure S5**). IHL were isolated at 7 dpi from wild-type and TSC1^flox^ mice infected with AdCre, followed by metformin treatment *in vitro*. The expression of p-S6 in T cells was significantly decreased in WT mice by metformin treatment in a dose-dependent manner; however, the loss of function in the TSC1^flox^ mice prevented the effect of metformin. Data also showed TSC1 deficiency increased p-S6 in both CD4 and CD8 T cells (**Figures 4A, B**). Our data suggest that metformin exhibited inhibitory effects on mTORC1 pathway in T cells following viral infection through the PI3K/TSC axis.

### Metformin Modulates Mitochondrial Fission in T Cells

**Figure 5:**
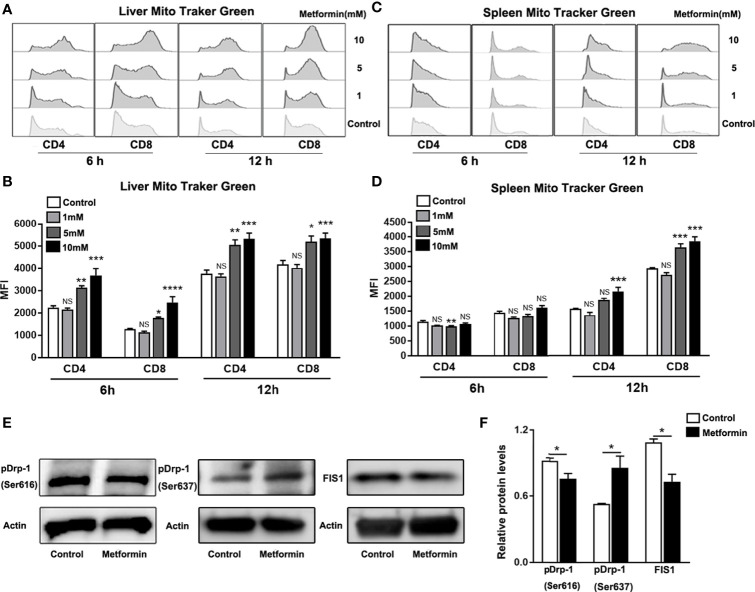
Metformin modulates mitochondrial fission in T cells. Mice were injected i.v. with 1.8 × 10^9^ pfu of AdLacZ and sacrificed at 7 dpi. T cells from liver and spleen were isolated and cultured with the indicated concentrations of metformin (0, 1, 5 and 10 mM) for 6 and 12 h. **(A)** Representative images of Mito Tracker Green histogram in IHL. **(B)** MFI of Mito Tracker Green in IHL. **(C)** Representative images of Mito Tracker Green histogram in splenic lymphocytes. **(D)** MFI of Mito Tracker Green in splenic lymphocytes. **(E, F)** western blot and statistical analysis of p-Drp-1(Ser616), Drp-1(Ser637) and FIS1 expression in CD8^+^ T cells. Data are representative of at least three independent experiments. Values are shown as mean ± SEM of n = 3-4 samples/group from single experiments representative of at least three experiments performed. One-way ANOVA with Tukey’s multiple comparisons test was used. Metformin treatment groups (1, 5 and 10 mM) were compared to the control group (0 mM). *p < 0.05; **p < 0.01; ***p < 0.001; ****p < 0.0001; ns, no significant difference.

T cell proliferation and function are closely associated with its metabolism process (33, 34). Mitochondria are the most important organelle with regards to cell metabolism (35, 36). Importantly, mitochondrial fission and fusion variation can make a flexible mitochondrial mass change depending on cell status (37, 38). For example, effector T cells have less mitochondrial mass than memory T cells, suggesting that mitochondria in effector T cells are actively undergoing fission while in memory T cells, these organelles exist in a fused state (39, 40). Furthermore, it was reported that metformin can target mTOR and contributes to normalizing mitochondrial function in T cells (41). We speculated that metformin acts on mitochondrial function in T cells. To test our hypothesis, we isolated IHL and splenocytes from Ad-infected mice and incubated these cells with metformin for 6 and 12 h. Using a mitochondrial tracker, we found a metformin treatment-induced increase of mitochondrial mass in IHL (**Figures 5A, B**) and splenocytes (**Figures 5C, D**). Dynamin-related protein-1(Drp-1) is a cytosolic/mitochondrial outer membrane protein, which is crucial for mitochondrial fission (40, 42). When phosphorylated at Ser616, Drp-1 stimulates mitochondrial fission. Conversely, fission is inhibited when DRP1 is phosphorylated at Ser637 (40, 43). To confirm this, we measured the p-Drp-1 (Ser616 and Ser637). As we expected, the expression of p-Drp-1 (Ser616) was decreased, while p-Drp-1 (Ser637) was increased by metformin treatment in splenic CD8^+^ T cells compared with that in the control (**Figures 5E, F**). Mitochondrial fission 1 protein (FIS1) is a protein that promotes mitochondrial fission. We found that metformin treatment also reduced the expression of FIS1 (**Figures 5E, F**). Therefore, our results indicate that metformin may regulate T cell responses *via* modulating mitochondrial fission.

### Metformin Promotes Mitochondrial Superoxide Production in T Cells

**Figure 6:**
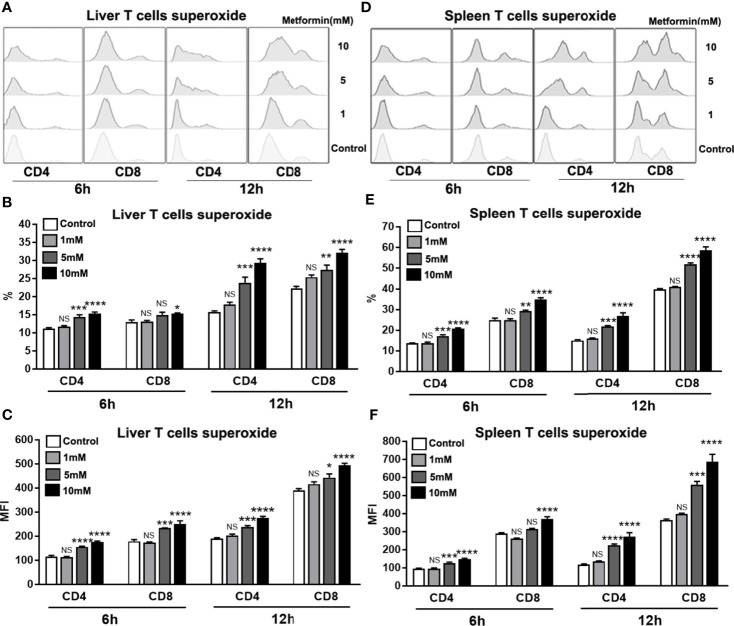
Metformin promotes mitochondrial superoxide production in T cells. Mice were injected i.v. with 1.8 × 10^9^ pfu of AdLacZ and sacrificed at 7 dpi. Lymphocytes from liver and spleen were isolated and cultured with the indicated concentrations of metformin (0, 1, 5 and 10 mM) for 6 and 12 h. Cells were collected and stained with MitoSOX Red mitochondrial superoxide indicator. **(A)** Representative images of mitochondrial superoxide histogram in IHL. **(B)** Percentage of mitochondrial superoxide in IHL. **(C)** MFI of mitochondrial superoxide in IHL. **(D)** MFI of mitochondrial superoxide in IHL in splenic lymphocytes. **(E)** Percentage of mitochondrial superoxide in splenic lymphocytes. **(F)** MFI of mitochondrial superoxide in splenic lymphocytes. Data are representative of at least three independent experiments. Values are shown as mean ± SEM of n = 3-4 samples/group from single experiments representative of at least three experiments performed. One-way ANOVA with Tukey’s multiple comparisons test was used. Metformin treatment groups (1, 5 and 10 mM) were compared to the control group (0 mM). *p < 0.05; **p < 0.01; ***p < 0.001; ****p < 0.0001; ns, no significant difference.

Mitochondria-derived reactive oxygen species (ROS) can mediate redox signaling, leading to aberrant T cell activation and increased cell apoptosis/death (43–46). Since metformin can target mitochondrial functions, we asked whether metformin can increase superoxide production in T cells. Using the MitoSOX Red mitochondrial superoxide indicator (47), we found that superoxide production was significantly augmented in T cells isolated from the liver (**Figures 6A–C**) and the spleen (**Figures 6D–F**) in a dose-dependent manner. Together, these data suggest that metformin may regulate T cell activity by promoting mitochondrial superoxide production.

## Discussion

It is well known that metformin is not only an anti-diabetic drug, but also has additional effects in treating cancer, obesity, NAFLD, polycystic ovary syndrome, and metabolic syndrome (1, 48–52). A clinical study also suggested that metformin has beneficial effects in patients with chronic hepatitis C (53), raising the possibility that metformin can be a therapeutic option for viral hepatitis. Indeed, metformin inhibits HCV infection *in vitro via* down-regulation of the mTOR signals (22, 54–56). In this study, we demonstrated that metformin treatment ameliorated liver injury in mice with viral hepatitis as evidenced by decreased serum ALT and AST levels, and alleviated liver pathological changes (**Figure 1**). Further analysis revealed that the attenuated liver injury by metformin treatment results from limited T cells responses in the liver (**Figure 2**). Metformin has been reported to orchestrate T cell responses in autoimmune diseases and transplant rejection (57, 58). We found that metformin treatment decreased the expression of TNF-α, which is a hepatocytotoxic mediator in the liver (24, 59). Our data highlight the immune-modulatory and anti-inflammatory roles of metformin in viral hepatitis. Notably, metformin treatment reduced the viral load in the liver (**Figure S1**), supporting a notion that metformin may contribute to antiviral responses, such as type I interferon (21–23).

Pharmacological effects of metformin are mediated by the activation of AMP-activated protein kinase (60), which negatively regulates the mTORC1 activity in T cells, leading to reduced cell proliferation and cytokine production (61–63). Here, we showed that metformin inhibited T cell activity as demonstrated by decreased IFN-*γ*, TNF-α and IL-2 expression (**Figures 3A, B**). The p-S6 levels in T cells were reduced significantly by metformin in a dose-dependent manner (**Figure 3C**). It is known that TSC1-TSC2 complex is a key negative upstream regulator of mTORC1 signaling (64). To confirm our *in vitro* results, we performed the experiment using T cells from AdCre infected-TSC1^flox^ mice, as we described previously (32). As expected, metformin failed to down-regulate p-S6 levels in TSC1 deficient T cells (**Figure 4B**). In all, our data demonstrated that metformin inhibited mTOR signals in T cells in a TSC1 dependent manner.

The mTOR signaling pathway plays a critical role in mitochondrial metabolism (65, 66). More recently, metformin has been shown to normalize mitochondrial function in T helper cells and alleviate aging-associated inflammation (41). This led us to investigate whether metformin contributes to mitochondrial function in T cells during viral hepatitis. To answer this question, we measured mitochondrial mass by flow cytometry (40) and demonstrated that mitochondrial mass was increased in T cells in a dose-dependent pattern by metformin (**Figures 5A–D**). It has been reported that naïve T cells display fused mitochondria (fusion) with higher mass, while effector T cells have mitochondria fission with lower mass (40). Our results suggest that metformin treatment inhibited mitochondrial fission and T effector function (**Figures 5A–D**). To confirm our conclusion that metformin modulates mitochondrial fission, we assessed expression of Drp-1 (Ser616), Drp-1 (Ser637) and FIS1, which are the key fission-associated proteins in effector T cells (40). Our data confirmed that metformin can modulate mitochondrial fission and fusion dynamics in T cells, leading to compromised T cell functions during viral infection.

T cell activation and proliferation are associated with dramatically increased bioenergetic, biosynthetic and redox demands (67–69). ROS, which are mainly produced by mitochondrial oxidative metabolism, contribute to T cell functions in diseases (70). Although moderate levels of ROS can act as key mediators within T cells to promote cell proliferation and clonal expansion, high levels of ROS result in T cell apoptosis through upregulation of Fas ligand, playing a necessary role in immune resolution (71). Moreover, increased ROS production in T cells facilitates the development of Th2 cells (72), while the inhibition of exogenous and endogenous ROS in T cells causes increased IFN-*γ* production (72, 73). We found that metformin treatment increased superoxide in activated T cells of infected mice (**Figure 6**). This result suggests that metformin may down-regulate excessive Th1 responses *via* induction of intrinsic ROS species, leading to better immune resolution and improved immunopathogenesis. In addition, metformin-induced superoxide may also contribute to T cell apoptosis (**Figure S3**) and limit inflammatory infiltration in the liver during acute viral hepatitis.

Together, this study has revealed for the first time a hepatoprotective role of metformin in acute viral hepatitis *via* mechanisms that include restraining excessive T cell infiltration and activation in the liver, inhibiting the mTOR signaling *via* a TSC1-dependent manner in T cells. Moreover, we have shown that metformin may contribute to mitochondrial fission and ROS production, leading to the orchestration of T cell-mediated immunity following viral infection. Overall, this study supports a beneficial role of metformin for viral hepatitis and also provides mechanistic evidence for the involvement of TSC1/mTOR in the regulation of T cell functions, providing a solid evidence for the therapeutic potential of metformin in viral infection.

## Data Availability Statement

The original contributions presented in the study are included in the article/**Supplementary Material**. Further inquiries can be directed to the corresponding authors.

## Ethics Statement

The animal study was reviewed and approved by the Institutional Animal Care and Use Committee (IACUC) of the University of Texas Medical Branch.

## Author Contributions

JS and YL conceived and designed the experiments. LX, XW, YL, and JS performed the experiments, analyzed the data, and wrote the paper. LS, JS, YoC, and JC contributed reagents. YaC, LS, and JC edited the paper. All authors contributed to the article and approved the submitted version.

## Conflict of Interest

The authors declare that the research was conducted in the absence of any commercial or financial relationships that could be construed as a potential conflict of interest.
