# Supplementary material for: Metformin Modulates T Cell Function and Alleviates Liver Injury Through Bioenergetic Regulation in Viral Hepatitis
Source: Front Immunol. 2021 Apr 21;12:638575. doi: 10.3389/fimmu.2021.638575 (PMC8097169; doi:10.3389/fimmu.2021.638575)
Supplement: Supplementary file 1 [file DataSheet_1.docx]

Supplementary Material

**Fig. S1. Metformin treatment facilitated viral clearance in the liver.** Mice were orally pretreated with metformin (250 mg/kg/day) for 1 week, followed by infection of adenovirus carrying LacZ (AdLacZ, 1.8 × 10^9^ pfu/mouse). Administration of metformin was continued for another 7 days. Normal salinewas used as a control. All mice were euthanized at 7 dpi. (A) Viral clearance was assessed by X-gal staining of frozen liver sections. Infected cells expressing X-gal activity were stained blue (3-5 mice per group), and (B) the percentages of blue area to whole photograph were calculated by Image J software. Data are representative of at least three independent experiments. Values are shown as mean ± SEM. Two-tailed unpaired T test was used for statistical analysis. * *p* < 0.05; ** *p* < 0.01.

**Fig. S2. Metformin treatment resulted in decreased numbers of activated T cells *in vivo*.** C57BL/6 mice were orally pretreated with metformin (250 mg/kg/day) for 1 week, followed by infection of AdLacZ (1.8 × 10^9^ pfu/mouse). Administration of metformin was continued for another 7 days. Normal saline was used as a control. All mice were euthanized at 7dpi. (A) Representative images of CD44^+^CD62L^-^ T cells. (B) Percentages and numbers of CD44^+^CD62L^-^ T cells. Data are shown as mean ± SEM of n = 3-5 mice/group from single experiments representative of at least three experiments performed. Two-tailed unpaired T test was used for statistical analysis. * *p* < 0.05; ** *p* < 0.01. ns, no significant difference.

**Fig. S3. Metformin treatment for 6 and 12 hours *in vitro* did not affect cell viability.** Mice were injected i.v. with 1.8 × 10^9^ pfu of AdLacZ and sacrificed at 7dpi. Lymphocytes were isolated from the liver and cultured with metformin *in vitro* (0, 5 and 10 mM) for 6, 12 and 24 h. Fixable Viability Dye was used to evaluate cell viability. (A) Representative flow cytometry dot plot of live cells. (B) Percentages of live cells after metformin treatment. Data are representative of at least three independent experiments. Values are shown as mean ± SEM. Two-tailed unpaired T test was used for statistical analysis. ** *p* < 0.01. ns, no significant difference.

**Fig. S4. Metformin treatment reduced the activation of splenic T cells *in vitro*.** Mice were injected i.v. with 1.8 × 10^9^ pfu of AdLacZ and sacrificed at 7dpi. T cells were isolated from spleens and cultured with metformin (0, 5 and 10 mM) for 6 and 12 h. Cells were stimulated with PMA and ionomycin in the presence of Golgi Stop during the last 5 h, followed by intracellular staining of IFN-γ, TNF-α and IL-2. (A) Percentages of IFN-γ^+^ TNF-α^+^ T cells. (B) Percentages of IFN-γ^+^ IL-2^+^ T cells. Data are representative of at least three independent experiments. Values are shown as mean ± SEM. Two-tailed unpaired T test was used for statistical analysis. * *p* < 0.05; ** *p* < 0.01; *** *p* < 0.001. ns, no significant difference.

**Figure S5. Expression of TSC1 in AdCre-infected mice.** TSC1^flox^ (TSC1^Δ^) and control mice were injected i.v*.* with 1.8 × 10^9^ pfu of AdCre. The transcript levels of liver TSC1 were analyzed. Values are shown as mean ± SEM. Two-tailed unpaired T test was used for statistical analysis. **** *p* < 0.0001.
