# Supplementary figures and images for: Metformin Modulates T Cell Function and Alleviates Liver Injury Through Bioenergetic Regulation in Viral Hepatitis
Source: Front Immunol. 2021 Apr 21;12:638575. doi: 10.3389/fimmu.2021.638575 (PMC8097169; doi:10.3389/fimmu.2021.638575)

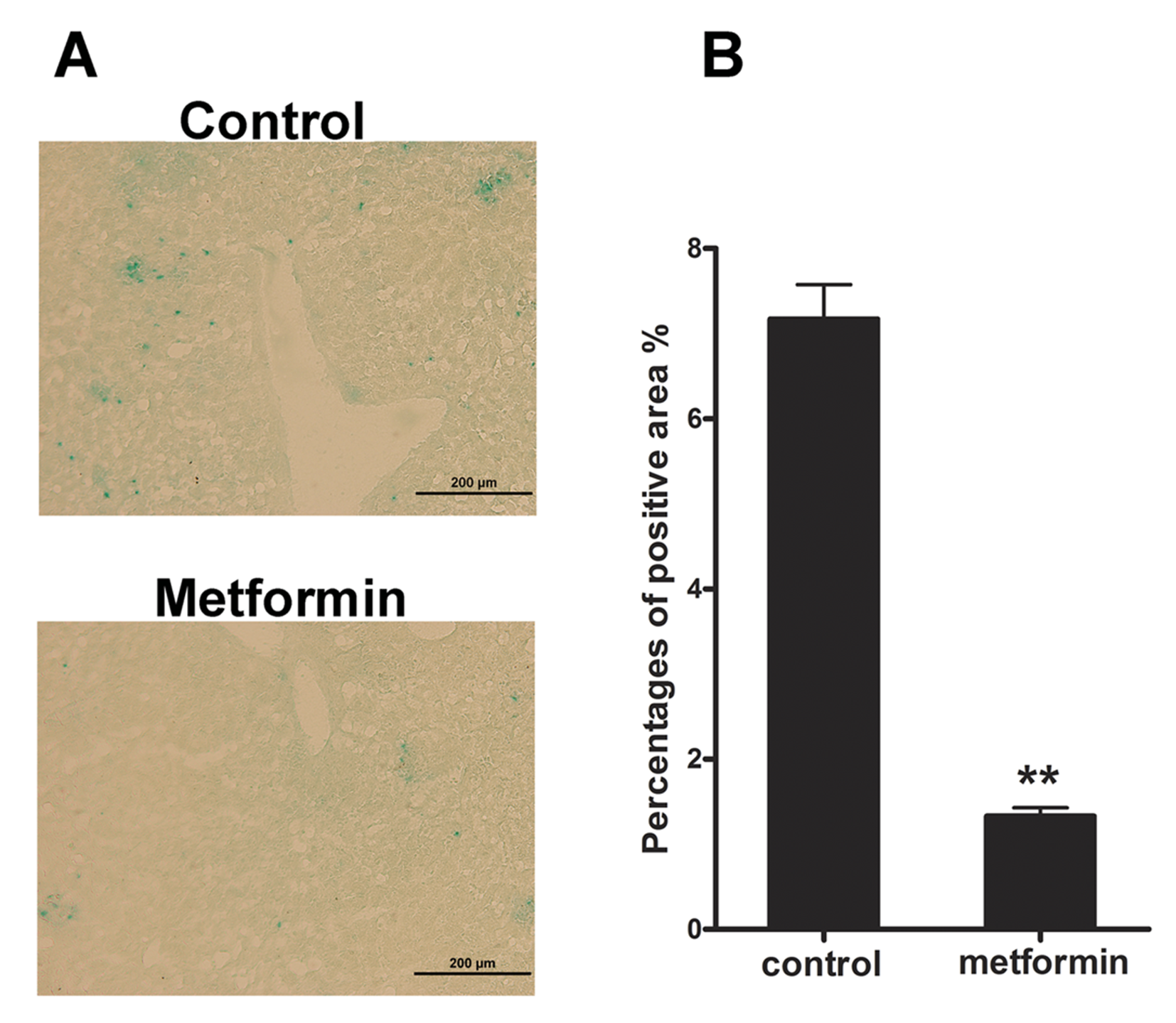

Supplement: Supplementary file 2 [file Image_1.tif]

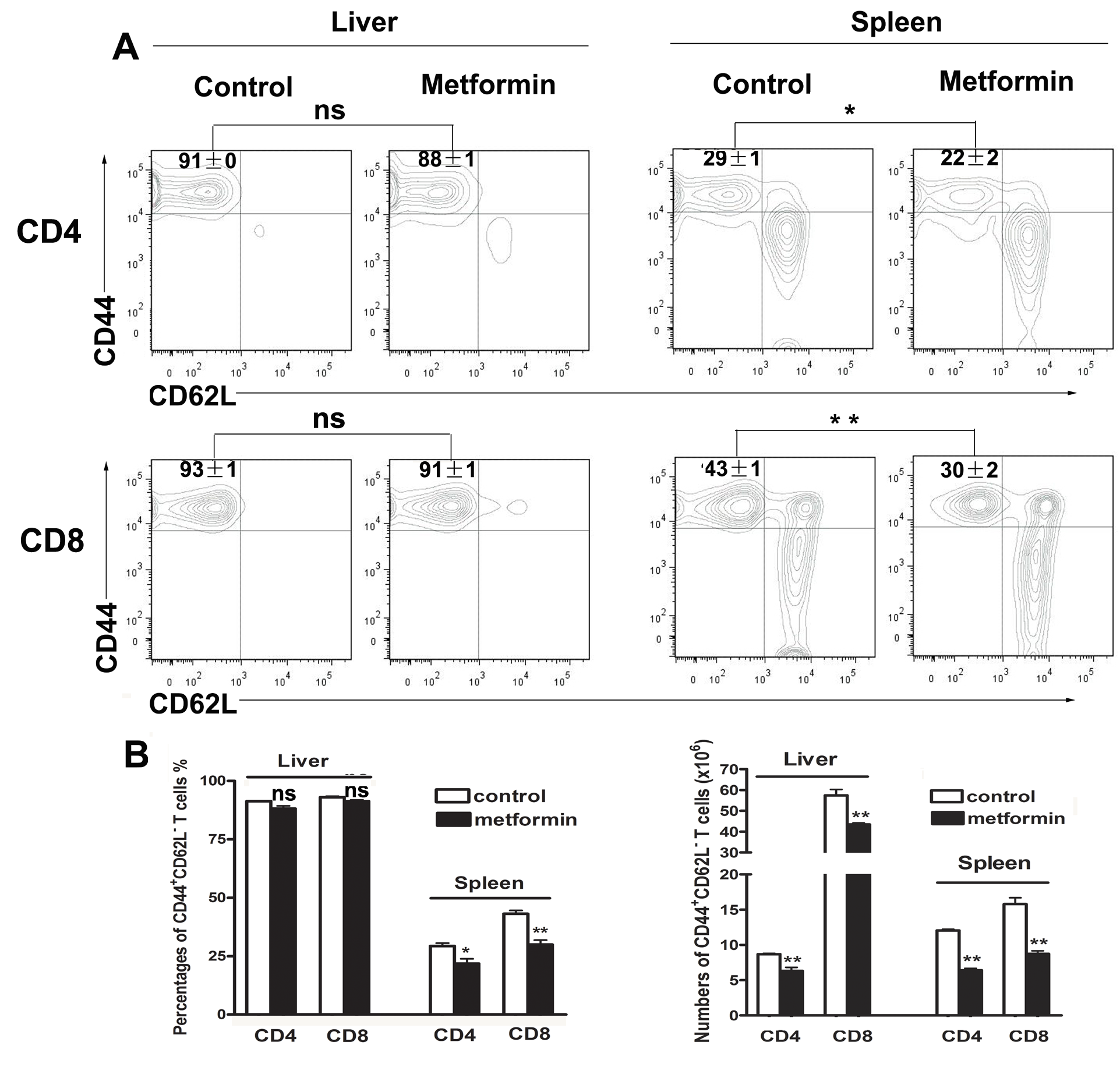

Supplement: Supplementary file 3 [file Image_2.tif]

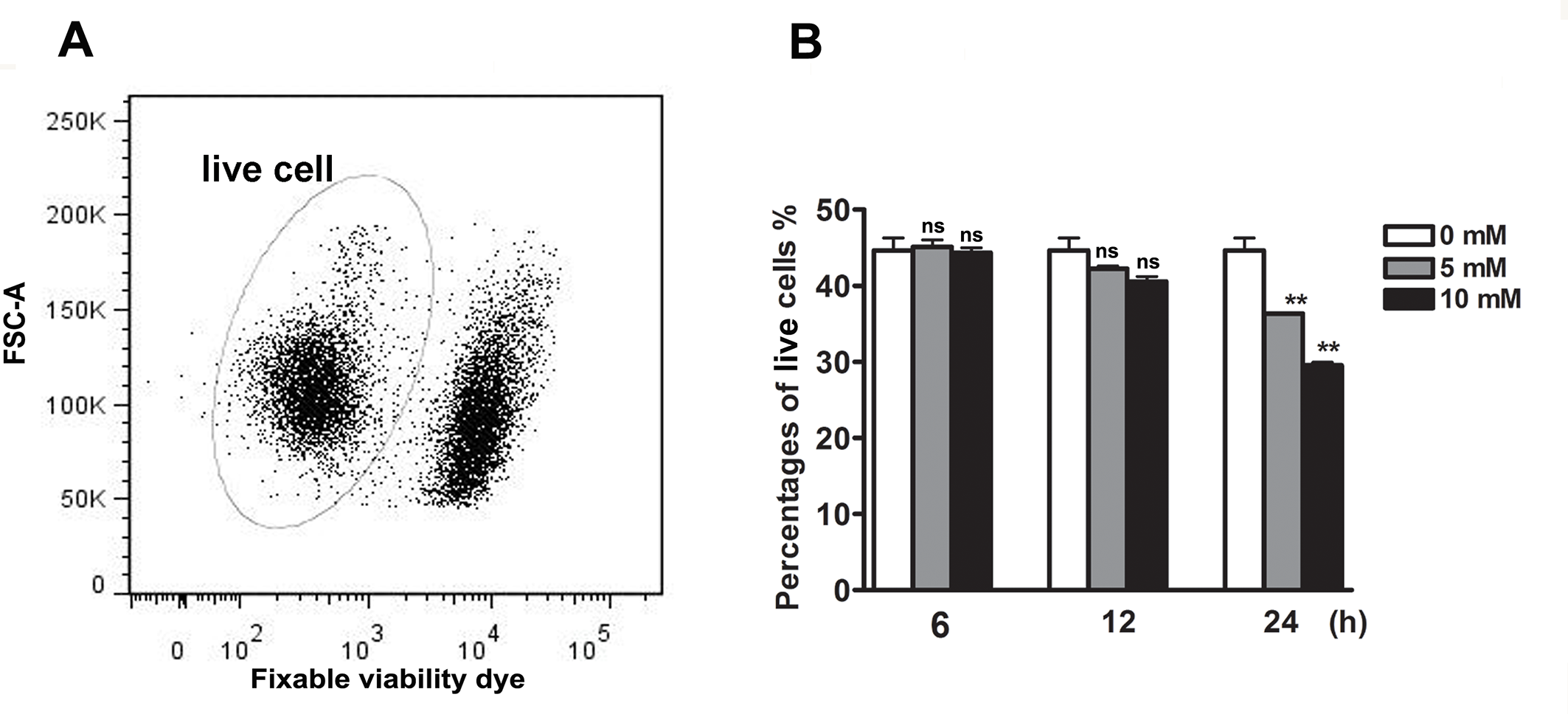

Supplement: Supplementary file 4 [file Image_3.tif]

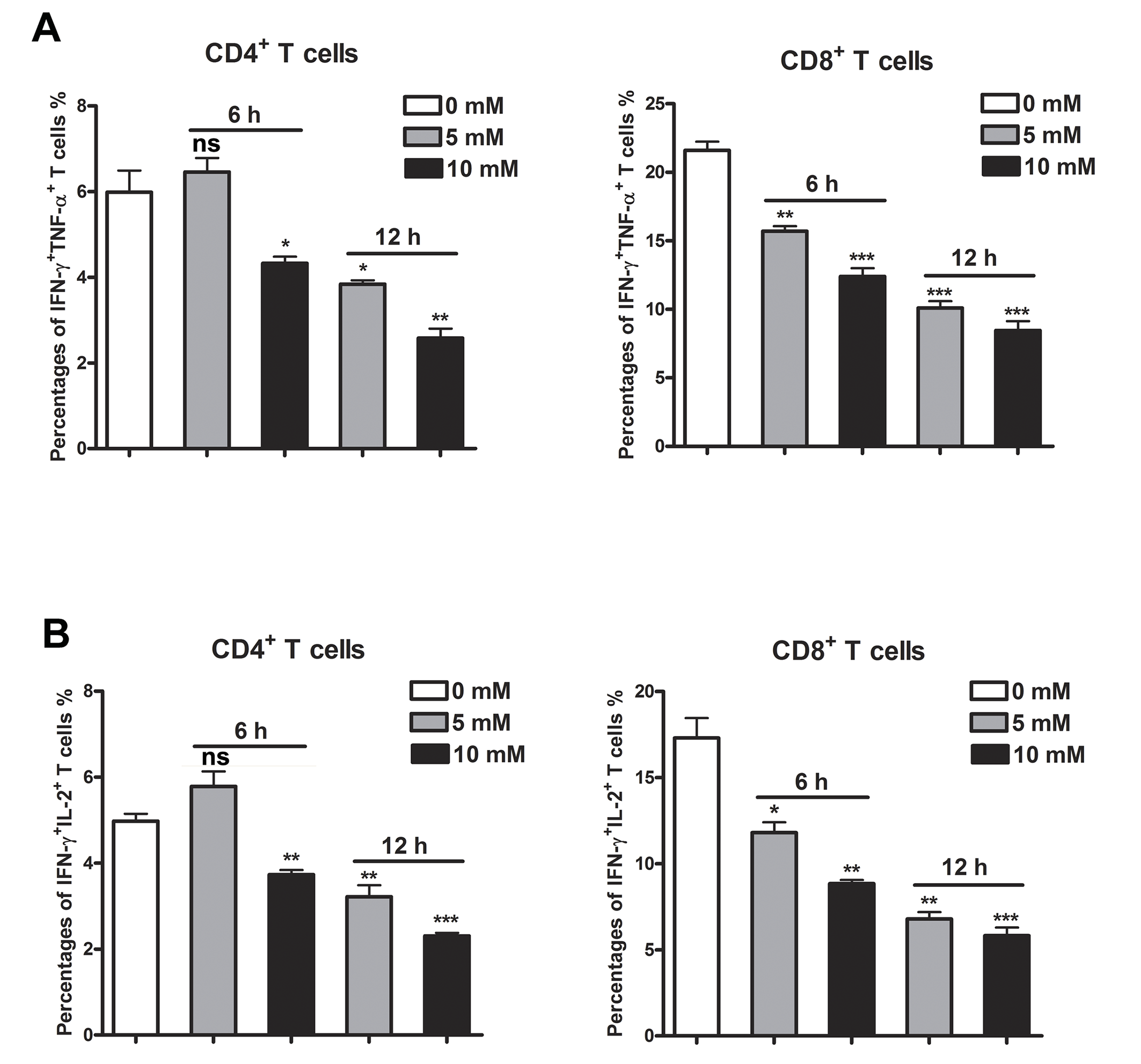

Supplement: Supplementary file 5 [file Image_4.tif]

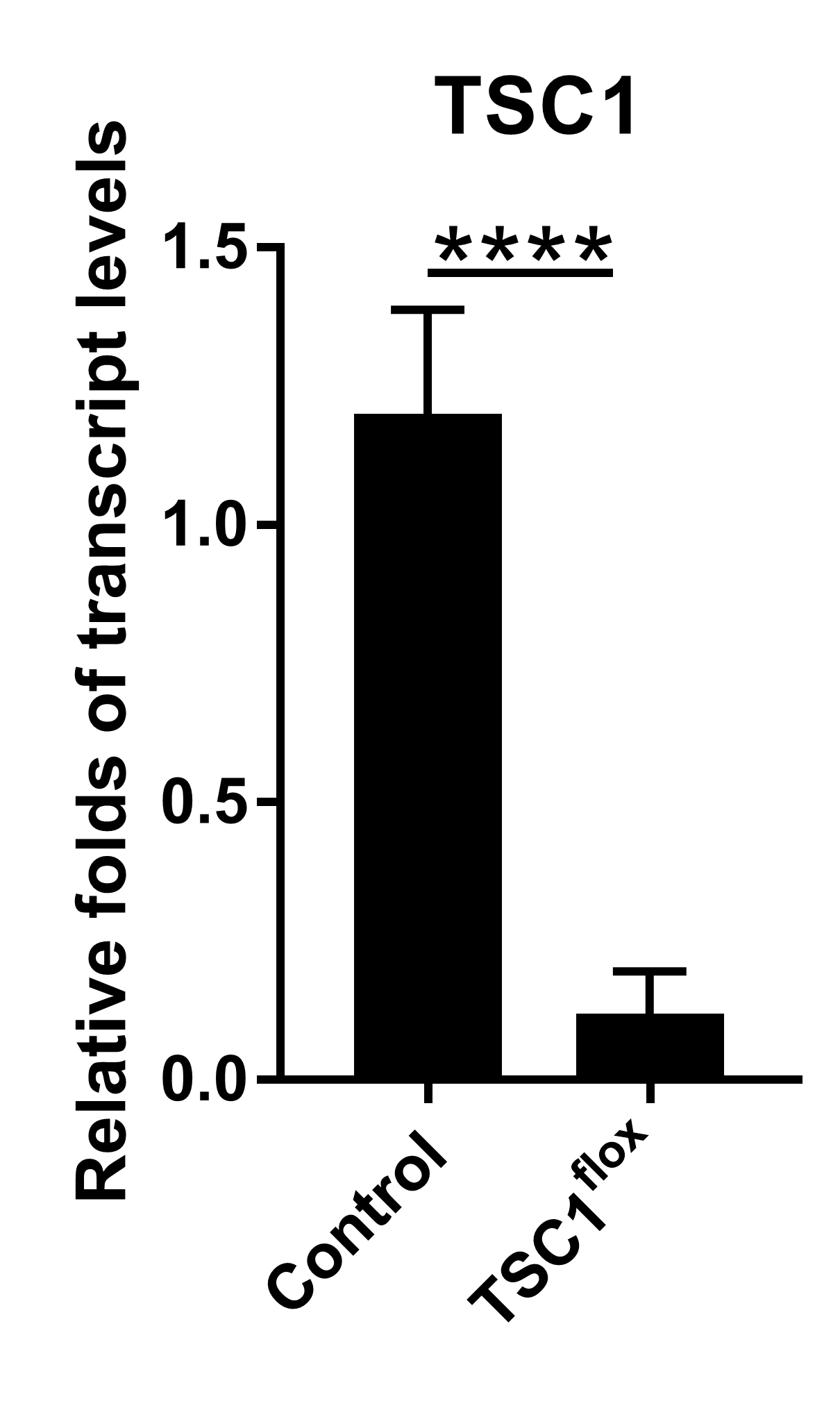

Supplement: Supplementary file 6 [file Image_5.tif]
